# Interleukin-22 predicts severity and death in advanced liver cirrhosis: a prospective cohort study

**DOI:** 10.1186/1741-7015-10-102

**Published:** 2012-09-11

**Authors:** Bernd Kronenberger, Ina Rudloff, Malte Bachmann, Friederike Brunner, Lisa Kapper, Natalie Filmann, Oliver Waidmann, Eva Herrmann, Josef Pfeilschifter, Stefan Zeuzem, Albrecht Piiper, Heiko Mühl

**Affiliations:** 1 Medizinische Klinik 1, Klinikum der J.W. Goethe Universität, Theodor-Stern-Kai 7, D-60590 Frankfurt am Main, Germany; 2 Pharmazentrum Frankfurt, Klinikum der J.W. Goethe Universität, Theodor-Stern-Kai 7, D-60590 Frankfurt am Main, Germany; 3 Institut für Biostatistik und mathematische Modellierung, Klinikum der J.W. Goethe Universität, Theodor-Stern-Kai 7, D-60590 Frankfurt am Main, Germany

**Keywords:** Interleukin-22, Liver cirrhosis, Liver-related complications, Hepatitis, Alcoholic liver disease, MELD

## Abstract

**Background:**

Interleukin-22 (IL-22), recently identified as a crucial parameter of pathology in experimental liver damage, may determine survival in clinical end-stage liver disease. Systematic analysis of serum IL-22 in relation to morbidity and mortality of patients with advanced liver cirrhosis has not been performed so far.

**Methods:**

This is a prospective cohort study including 120 liver cirrhosis patients and 40 healthy donors to analyze systemic levels of IL-22 in relation to survival and hepatic complications.

**Results:**

A total of 71% of patients displayed liver cirrhosis-related complications at study inclusion. A total of 23% of the patients died during a mean follow-up of 196 ± 165 days. Systemic IL-22 was detectable in 74% of patients but only in 10% of healthy donors (*P *< 0.001). Elevated levels of IL-22 were associated with ascites (*P *= 0.006), hepatorenal syndrome (*P *< 0.0001), and spontaneous bacterial peritonitis (*P *= 0.001). Patients with elevated IL-22 (>18 pg/ml, n = 57) showed significantly reduced survival compared to patients with regular (≤18 pg/ml) levels of IL-22 (321 days *versus *526 days, *P *= 0.003). Other factors associated with reduced overall survival were high CRP (≥2.9 mg/dl, *P *= 0.005, hazard ratio (HR) 0.314, confidence interval (CI) (0.141 to 0.702)), elevated serum creatinine (*P *= 0.05, HR 0.453, CI (0.203 to 1.012)), presence of liver-related complications (*P *= 0.028, HR 0.258, CI (0.077 to 0.862)), model of end stage liver disease (MELD) score ≥20 (*P *= 0.017, HR 0.364, CI (0.159 to 0.835)) and age (*P *= 0.011, HR 0.955, CI (0.922 to 0.989)). Adjusted multivariate Cox proportional-hazards analysis identified elevated systemic IL-22 levels as independent predictors of reduced survival (*P *= 0.007, HR 0.218, CI (0.072 to 0.662)).

**Conclusions:**

In patients with liver cirrhosis, elevated systemic IL-22 levels are predictive for reduced survival independently from age, liver-related complications, CRP, creatinine and the MELD score. Thus, processes that lead to a rise in systemic interleukin-22 may be relevant for prognosis of advanced liver cirrhosis.

## Background

Liver cirrhosis, virally-, drug- or alcohol-induced, is a major health problem associated with significant morbidity and mortality worldwide [1]. Identification of processes that lead to deterioration of liver cirrhosis and development of complications is regarded as key to successful implementation of novel treatment regimes aiming at hard-to-treat patients suffering from hepatitis of diverse etiologies.

Interleukin (IL)-22 is among newly identified parameters of hepatocyte biology that recently became the major focus of basic and translational research on liver injury and inflammation [2]. This member of the IL-10 cytokine family is primarily produced by activated CD4^+ ^or CD8^+ ^T cells, γδ-T cells, macrophages/dendritic cells and a diverse array of natural killer (NK)-like cells recently coined innate lymphoid cells [3-5]. Enhanced IL-22 expression is associated with diseases triggered by or accompanied with immunoactivation, among others: psoriasis [6], inflammatory bowel diseases [7], rheumatoid arthritis [8] and abdominal sepsis [9]. IL-22 is biochemically and functionally akin to IL-6 and able to efficiently initiate the hepatic acute phase response [10,11]. However, in contrast to IL-6, IL-22 almost exclusively acts on non-leukocytic cells. As a result, cells of epithelial origin, including hepatocytes, but not leukocytes, are major targets of IL-22 [3-5]. Pathophysiological functions of IL-22 as detected in rodent disease models are strictly context specific. In fact, this cytokine appears to be pathogenic in models of diseases associated with autoimmunity and tissue hyperplasia, such as experimental psoriasis [12] and arthritis [13]. In contrast, IL-22 has protective functions in models of microbe/infection-driven inflammation at host/environment interfaces [14-16], likely by up-regulating anti-microbial peptides [3-5,14,15], inducible nitric oxide (NO) synthase [17,18] and mucus production [16]. The tissue protective properties of IL-22 also extend to experimental ventilator-induced lung injury [19] and, in particular, to specific models of hepatic diseases [5].

As already alluded to, the liver is regarded as a prime target of IL-22 biological activity. Likely by promoting hepatocyte survival, several studies indicate a protective role for IL-22 in experimental hepatitis and liver injury [20-22], a property that may on the other hand promote progression to hepatocellular carcinoma [23]. However, the role of IL-22 in liver disease is not unequivocal. A recent report indicates that IL-22 may also amplify liver injury in experimental hepatitis B virus infection [24]. Notably, the course of hepatic disease in this context depends on robust leukocyte infiltration, which is at least in part dependent on IL-22 [24]. The potential of IL-22 as a parameter of liver diseases is further highlighted by detection of its increased expression in patients' liver biopsy specimens [23,25,26]. Enhanced levels of systemic IL-22 have recently been observed in patients with chronic hepatitis [23] and acute hepatitis B infection [24].

Liver cirrhosis, the end-stage of various liver diseases, has a poor prognosis, which is determined by deterioration of hepatic functional capacity and consecutive development of hepatic complications. In the cirrhotic liver, IL-22 may be secreted to protect residual healthy liver tissue. Assuming that IL-22 possesses hepatoprotective properties in end-stage liver disease, IL-22 may be a relevant factor for progression of liver cirrhosis. The prognostic relevance of elevated IL-22 levels in patients with end-stage liver disease has not been characterized so far. Here we comprehensively analyzed systemic IL-22 concentrations in a cohort of 120 patients suffering from severe liver cirrhosis and thoroughly related those data to the survival of liver disease.

## Methods

### Study population and selection of patients

This prospective study investigated complications and mortality in consecutive patients who were treated due to advanced liver cirrhosis at the liver center of the University Hospital Frankfurt, Germany. The study protocol was approved by the local ethics committee of the University Hospital Frankfurt, Germany (reference number 84/09). All patients gave written informed consent before inclusion into the study. The study was performed in accordance with the Declaration of Helsinki.

Inclusion criteria were age ≥18 years and the presence of liver cirrhosis. Liver cirrhosis was either proven histopathologically or by explicit morphological criteria of liver cirrhosis with ultrasound, computer tomography or magnetic resonance imaging. Exclusion criteria were patients with organ transplantation and patients with early to terminal stages of hepatocellular carcinoma. Patients with diagnosis of hepatocellular carcinoma after study inclusion were not excluded later on. The control group consisted of healthy volunteers (n = 40, 32.4 years ± 8.4 years).

### Evaluation of complications

The following forms of liver-related complications were investigated during hospital admission: ascites, spontaneous bacterial peritonitis, hepatorenal syndrome, esophageal variceal bleeding and hepatic encephalopathy. The presence of ascites was confirmed by ultrasonography. Paracentesis was performed if appropriate amounts of ascites were detectable. Duplex sonography of the liver veins as well as of the portal vein was performed each time ascites was present. When Budd Chiari syndrome, portal vein thrombosis or a malignant ascites was diagnosed at first admission, the patients were excluded. The diagnosis of spontaneous bacterial peritonitis was based on a neutrophil count >250 mm³ in ascites fluid and/or a positive ascitic fluid culture. Hepatorenal syndrome was diagnosed according to the EASL clinical practice guideline [1]. Type 1 hepatorenal syndrome was diagnosed when the serum creatinine level was increased by more than 100% above baseline to a final level >2.5 mg/dl during the first hospitalization. Esophageal variceal bleeding was diagnosed by endoscopy. Hepatic encephalopathy was diagnosed according to clinical criteria, including impairment of autonomy, consciousness, intellectual function and behavior.

### Study endpoints

The primary endpoint of this study was mortality in patients with advanced liver cirrhosis. Furthermore, the association between liver-related complications, such as ascites, hepatorenal syndrome, spontaneous bacterial peritonitis and hepatic encephalopathy with IL-22, was analyzed. In addition, the relation between IL-22 serum levels and laboratory parameters as well as the model of end stage liver disease (MELD) score was assessed.

### Collection of blood samples

All samples for IL-22 baseline quantification were collected at study inclusion. In hospitalized patients peripheral blood was collected within the first week of hospital admission. Furthermore, blood was collected in consecutive patients with liver cirrhosis coming to the outpatient clinic. In addition, blood was collected during the follow-up period. For follow-up analyses, blood was collected at subsequent hospitalizations after study entry. Follow-up serum samples were taken a minimum of 30 days apart from the initial blood collection. Blood samples were subsequently used for clinical chemistry and IL-22 quantification. Serum tubes were centrifuged at 1,500 × g for 10 minutes at 4°C, followed by an additional centrifugation step at 2,000 × g at 4°C to completely remove any remaining cells. The serum samples were aliquoted and stored at -80°C until further use.

### Clinical chemistry

Hematology and biochemistry analyses were performed at the local laboratory of the Frankfurt University Hospital.

### Quantification of IL-22 serum levels

Serum IL-22 levels were quantified by using the IL-22 Quantikine enzyme linked immunosorbent assay (ELISA) according to the manufacturer's instructions. This assay detects a minimum of 2.7 pg/ml of IL-22 (R&D Systems, Inc., Minneapolis, MN, USA). All samples showing a value below this detection limit were set to 2.6 pg/ml. Readers of the IL-22 ELISA were unaware of the patients' clinical characteristics.

### IL-22 immunohistochemistry

Liver biopsies were performed using a 14-gauge modified Menghini needle under ultrasound guidance and local anesthesia. Liver biopsy specimens were fixed in formalin and embedded in paraffin for routine staining with hematoxylin and eosin. For immunohistochemical staining, 10 μm paraffin sections were deparaffinized through xylene and graded ethanol. For antigen retrieval, paraffin sections were boiled in citrate buffer (10 mM, pH 6.0) for 30 minutes. Endogenous peroxidases were blocked by incubation in a solution containing 3% H_2_O_2 _in methanol for 10 minutes. IL-22 was detected by incubating the tissue sections with a monoclonal anti-human anti-IL-22 antibody (clone ab50140, Abcam, Cambridge, UK) for 1 h (dilution of 1:75) in humidified atmosphere. Thereafter, the tissue slices were incubated with horseradish peroxidase-labeled polymer conjugated goat anti-mouse antibody (EnVision™, Dako, Hamburg, Germany) for 30 minutes. Diaminobenzidin was used as chromogen.

### Statistical analyses

The follow-up period started at study inclusion and ended at the patient's time of death, liver transplantation or last contact. Death was recorded as an event. All patients were seen every 4 to 12 weeks at the liver center of the J.W. Goethe University Hospital. At the end of the study, the patient's physician was contacted. In patients who were lost to follow-up before the end of the study, the time in the study ranged from study inclusion to last contact. Statistical analyses were performed with SPSS (Version 17.0, IBM, New York, NY, USA) and BiAS (Epsilon Verlag, Hochheim Darmstadt, Germany). Overall survival rates were assessed for all patients with the Kaplan-Meier method and compared with the log-rank test. Correlations between two variables were assessed by the non-parametric Spearman test.

The MELD score was calculated according to the standard formula as follows: 11.2 × ln (INR) + 9.57 × ln (creatinine, in milligrams per deciliter) + 3.78 × ln (bilirubin, in milligrams per deciliter) + 6.43 with a lower limit of 1 for all variables and with creatinine capped at 4. A multivariate Cox proportional-hazards analysis using forward stepwise variable selection was conducted to describe the relationship between the systemic IL-22 serum level, the MELD score, CRP, age, creatinine, presence of liver-related complications and overall survival. Furthermore, an area under the observed receiver operating curve (AUC) analysis was performed to compare the performance of elevated IL-22 and the MELD score for the prediction of survival.

Categorical variables were analyzed using the Fisher test. Differences between paired and unpaired samples were investigated with the non-parametric Wilcoxon paired sample test, the Mann-Whitney U-test or the Kruskal Wallis test. All tests were two-sided and a *P*-value of < 0.05 was considered statistically significant.

## Results

### Patient characteristics

**Figure 1:**
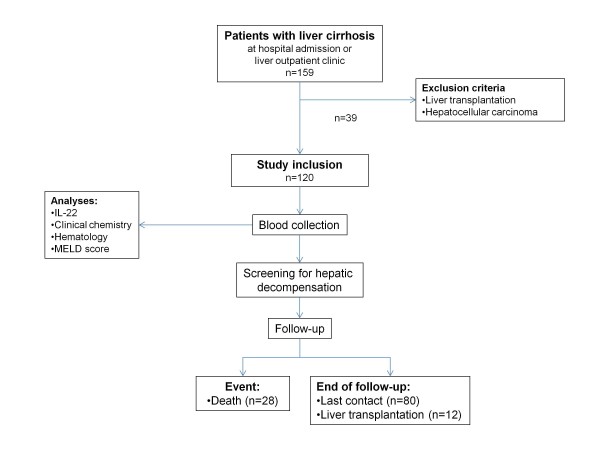
**Study protocol**.

Between 2009 and 2011, 159 patients with liver cirrhosis were screened. A total of 120 patients fulfilled the inclusion criteria and were prospectively enrolled into the present study (Figure 1). The patient characteristics are shown in Table 1. The majority of patients was male. The major causes of liver cirrhosis were alcoholic liver damage, chronic viral hepatitis B or C, respectively. The majority of patients presented with liver cirrhosis related complications at inclusion into the study. The most frequent liver-related complication was ascites. Among patients with ascites, 19.1% had spontaneous bacterial peritonitis and 32.4% hepatorenal syndrome. Criteria for hepatorenal syndrome type 1 and 2 were fulfilled in 12 and 14 patients, respectively.

**Table 1:** Patient characteristics

Parameter	
**Patients, n**	120
Male, n (%)	77 (64.2)
Age, mean ± SD (years)	56.1 ± 11.8
Weight, mean ± SD (kg)	77.8 ± 17.0
	
**Hematology**	
Hb, mean ± SD (g/dl)	11.1 ± 2.8
Leukocytes, mean ± SD (10³)	6.8 ± 4.1
Thrombocytes, mean ± SD (/nl)	134.5 ± 167.3
	
**Biochemistry**	
AST, mean ± SD (U/l)	74.9 ± 116.5
ALT, mean ± SD (U/l)	55.1 ± 150.5
GGT, mean ± SD (U/l)	186.6 ± 209.6
AP, mean ± SD (U/l)	148.2 ± 82.0
Total bilirubin, mean ± SD (mg/dl)	3.6 ± 5.6
INR, mean ± SD	2.3 ± 8.2
CRP, mean ± SD (ng/ml)	2.3 ± 3.1
Albumin, mean ± SD (g/l)	3.2 ± 0.6
Creatinine, mean ± SD (mg/dl)	1.3 ± 0.9
	
**Prognosis score**	
MELD, mean (range)	16.3 (6.2 to 35.6)
	
**Liver-related complications**	
Ascites, n (%)	68 (56.7)
Hepatorenal syndrome, n (%)	26 (21.7)
Spontaneous bacterial peritonitis, n (%)	13 (10.8)
Hepatic encephalopathy, n (%)	25 (20.8)
Variceal bleeding, n (%)	10 (8.3)
	
**Etiology of liver disease**	
Chronic hepatitis B, n (%)	10 (8.3)
Chronic hepatitis C, n (%)	34 (28.3)
Alcohol, n (%)	54 (45.0)
PSC, n (%)	4 (3.3)
PBC, n (%)	2 (1.7)
Autoimmune, n (%)	3 (2.5)
Hemochromatosis n (%)	2 (1.7)
NASH, n (%)	2 (1.7)
Toxic, n (%)	2 (1.7)
Alpha1 antitrypsin deficiency	1 (0.8)
Cryptogenic	6 (5.0)

Normal reference ranges for male and female patients, respectively, were 13.7 to 17.5 and 11.2 to 15.7 g/dl for hemoglobin (Hb), 4.0 to 10.4 and 4.2 to 9.07/nl for leukocytes, 163 to 337 and 182 to 369/nl for thrombocytes, 3.5 to 5.2 and 3.5 to 5.2 g/l for albumin. Upper limits of normal for male and female patients, respectively, were 40 and 35 U/l for aspartate aminotransferase (AST), 50 and 35 U/l for alanine aminotransferase (ALT), 60 and 40 U/l for gamma glutamyl transpeptidase (GGT), 130 and 105 U/l for alkaline phosphatase (AP), 1 and 1 mg/dl for total bilirubin, 0.5 and 0.5 ng/ml for C reactive protein (CRP), 1.25 and 1.09 mg/dl for creatinine. MELD, Model of end stage liver disease; PBC, primary biliary cirrhosis; PSC, primary sclerosing cholangitis; SD, standard deviation.

The patients were followed for a mean and maximum duration of 195.7 ± 165.1 days and 651 days, respectively. Twenty-eight patients (23.3%) died during the follow-up period. All cases of death were considered as related to deterioration of liver cirrhosis and complications. In two patients, hepatocellular carcinoma was diagnosed after inclusion into the study. Twelve patients (10%) received liver transplantation during the observation period.

### IL-22 serum levels are increased in patients with liver cirrhosis

**Figure 2:**
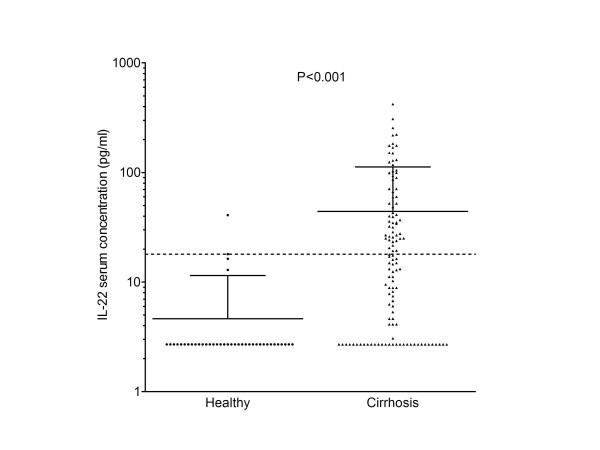
**IL-22 serum concentrations are elevated in patients with liver cirrhosis (n = 120) compared with healthy individuals (n = 40)**. Dots indicate IL-22 serum levels in individual patients. The straight horizontal line indicates the mean. The dotted horizontal line indicates the upper limit of normal for IL-22 of 18 pg/ml. Error bars indicate the standard deviation. Comparison between the two groups was performed using the Mann Whitney U-test.

To investigate whether liver cirrhosis is associated with increased serum IL-22, the cytokine was determined in sera of healthy donors and liver cirrhosis patients, respectively. IL-22 was detectable (>2.6 pg/ml) in 89 of 120 patients with liver cirrhosis, but only in 4 of 40 healthy controls (74.1% vs. 10.0%, *P *< 0.001) (Figure 2). The corresponding mean levels of serum IL-22 as detectable by ELISA were 10-fold higher in patients with liver cirrhosis than in healthy donors (44.1 ± 68.4 and 4.6 ± 6.8 pg/ml, Figure 2). The control group in this study was younger than the patient cohort (32.4 years vs. 56.1 years). To exclude a potential bias we investigated if age and gender may influence the IL-22 serum concentration. Age and gender were not associated with the systemic IL-22 level in patients with liver cirrhosis (*P *> 0.2 for both). Furthermore, the mean value of systemic IL-22 in healthy controls obtained in this study was similar to the mean systemic level of IL-22 in the literature reported for older patients (3.3 ± 1.4 pg/ml, 51.6 ± 7.6 years) [27].

In order to determine a reference range, we used the 95% interval of lL-22 serum concentrations that were observed in healthy donors. Based on that strategy, we defined the upper limit of normal (ULN) serum IL-22 concentration to be 18 pg/ml. According to this ULN, 57 out of 120 (47.5%) patients with liver cirrhosis but only 2 out of 40 (5.0%) healthy donors displayed elevated IL-22 serum levels.

### IL-22 serum levels increase in the course of liver disease

**Figure 3:**
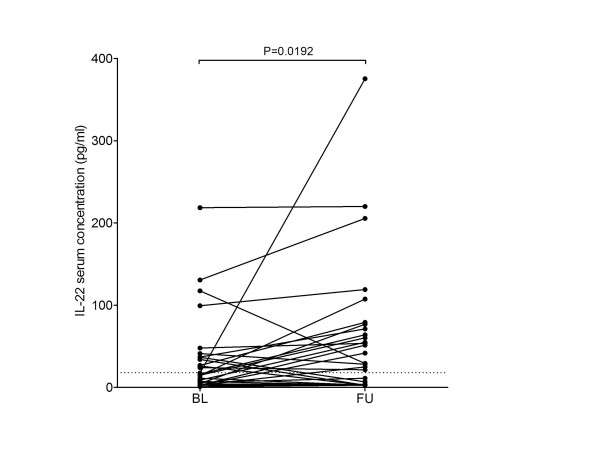
**Changes of IL-22 in the course of liver disease**. Dots indicate serum IL-22 levels in individual patients at baseline (BL) and follow-up (FU), respectively. Corresponding IL-22 serum levels in individual patients at BL and FU are connected. IL-22 serum levels were analyzed in follow-up (FU) sera that were a minimum of 30 days apart from baseline (BL). The dotted horizontal line indicates the upper limit of normal for IL-22 of 18 pg/ml. Comparison between BL and FU was performed with the Wilcoxon matched pairs test.

Next, we were interested whether IL-22 serum levels are stably increased in the course of liver disease. Follow-up sera were available in 29 patients with liver cirrhosis. Thirteen patients (44.8%) had elevated IL-22 serum levels at baseline. After a mean follow-up of 138.2 ± 114.0 days, the mean IL-22 serum level increased from 33.3 ± 49.2 pg/ml to 61.3 ± 82.2 pg/ml (*P *= 0.0192, Figure 3). Only 3 of 13 patients (23.1%) with elevated IL-22 serum levels at baseline showed a decline of IL-22 below the ULN at follow-up while 9 of 16 patients (56.3%) with IL-22 serum levels below the ULN at baseline showed an increase of IL-22 above ULN at follow-up.

### IL-22 is detectable in livers from patients with liver cirrhosis

**Figure 4:**
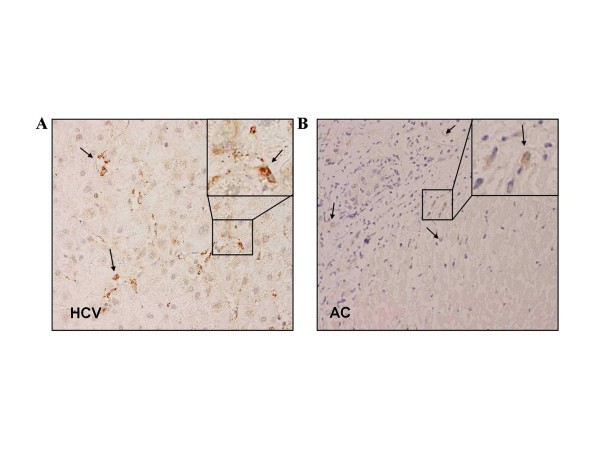
**Immunohistochemical detection of IL-22 in the liver**. IL-22 positive cells were non-hepatocytic cells. IL-22 positive cells were detected in cirrhotic livers with different etiologies. HCV, chronic hepatitis C (**A**); AC, alcoholic cirrhosis (**B**); arrows indicate typical IL-22 positive cells which were different from hepatocytes. The inset shows a magnification of IL-22 positive cells.

A recent report demonstrates that IL-22 is produced locally in livers of patients with chronic viral hepatitis [26]. In order to provide evidence that enhanced systemic IL-22 as observed herein likely derived from diseased liver tissue, IL-22 expression was determined in liver biopsies by immunohistochemical staining (available from only 10 patients, as liver biopsies are not routinely performed in patients with advanced liver cirrhosis). IL-22 positive cells were observed in 7 of 10 liver biopsies from patients with different etiologies of liver cirrhosis. In agreement with Park *et al. *[26], IL-22 expression was detectable mainly in non-parenchymal cells (Figure 4).

### Serum IL-22 and etiologies of liver disease

We next investigated whether distinct etiologies of liver diseases in the patient cohort under investigation affected IL-22 serum levels. Notably, no significant differences became apparent between levels of IL-22 in sera from patients with liver cirrhosis due to chronic hepatitis B (HBV), chronic hepatitis C (HCV) and alcoholic cirrhosis (AC) (*P *> 0.2). For hereditary, cholestatic, autoimmune liver diseases as well as toxic liver injury and non-alcoholic steatohepatitis, the number of patients was too low to draw a valid conclusion. Patients with chronic HBV, chronic HCV and alcoholic cirrhosis had significantly higher IL-22 serum levels than healthy controls (*P *= 0.009, *P *< 0.001 and *P *< 0.001, respectively). These data do not support an association between the etiology of the underlying liver disease and elevated serum IL-22.

### Elevated IL-22 serum levels are associated with reduced survival of patients with liver cirrhosis

**Figure 5:**
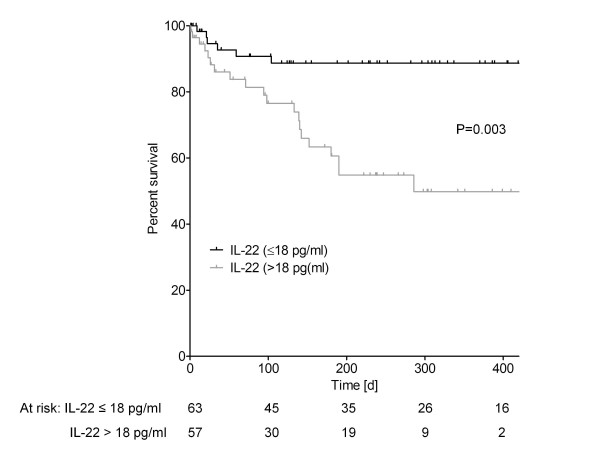
**Survival of patients with normal and elevated IL-22 serum levels**. Kaplan-Meier curve for survival of patients with normal (IL-22 ≤18 pg/ml, black line) and elevated IL-22 levels (IL-22 >18 pg/ml, grey line). Survival was significantly higher in patients with normal vs. elevated IL-22 serum levels according to the log rank test (*P *= 0.003). The number of patients at risk is shown in the table below the plot.

To investigate whether IL-22 serum levels are associated with survival of patients with liver cirrhosis, we compared survival of patients with liver cirrhosis and normal IL-22 levels (below the ULN of 18 pg/ml) with survival of patients having elevated IL-22 serum levels (above the ULN of 18 pg/ml). As illustrated in Figure 5, survival of patients with elevated IL-22 serum levels was significantly reduced compared to patients with normal IL-22 serum levels (*P *= 0.003). The estimated mean survival time was 526.4 days for patients with normal systemic IL-22 and 321.3 days for patients with elevated IL-22 (Figure 5).

### IL-22 serum levels are associated with complications of liver cirrhosis

**Figure 6:**
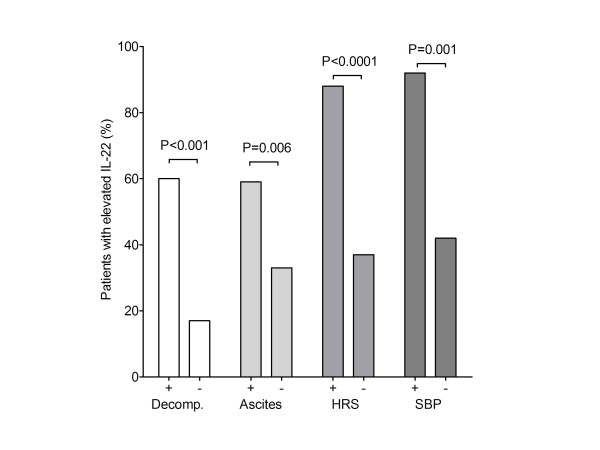
**Liver-related complications in patients with liver cirrhosis according to IL-22 serum levels**. Columns show the percentage of patients with elevated serum IL-22 above 18 pg/ml with (+) or without (-) liver related complications, ascites, hepatorenal syndrome (HRS), spontaneous bacterial peritonitis (SBP) at time of IL-22 quantification. Groups were compared by the Fisher test.

To investigate whether systemic IL-22 levels are associated with complications of liver cirrhosis, we compared liver cirrhosis-related complications between patients with IL-22 serum levels above or below the ULN of 18 pg/ml. Elevated IL-22 levels were more frequent in patients with liver cirrhosis-related complications than in patients with compensated liver cirrhosis (60.0% vs. 17.1%, *P *< 0.001). Moreover,elevated IL-22 serum levels were more frequent in patients with ascites, hepatorenal syndrome (HRS) and spontaneous bacterial peritonitis as compared to patients without these complications (Figure 6).

### IL-22 serum levels correlate with MELD score

**Figure 7:**
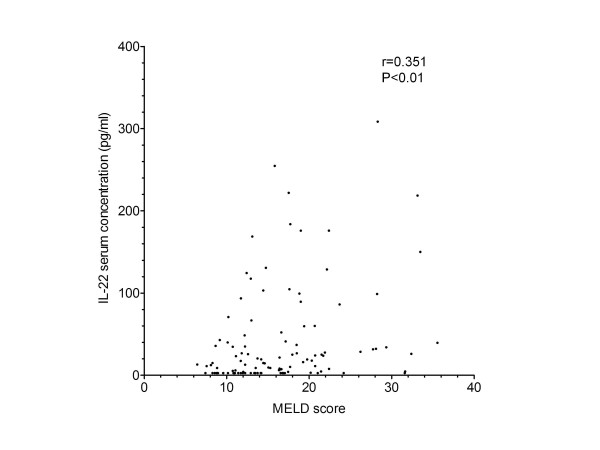
**Correlation between MELD score and IL-22 serum levels in patients with liver cirrhosis**. The correlation coefficient was calculated by the Spearman test.

The currently best-evaluated prognostic score for patients with liver cirrhosis is the MELD score. In the present study, there was a significant association between high MELD score (≥20) and reduced survival (*P *= 0.017, hazard ratio (HR) 0.364, confidence interval (CI) (0.159 to 0.835)). As IL-22 serum levels were associated with mortality of patients with liver cirrhosis, we investigated the relation between the MELD score and IL-22 serum levels. As shown in Figure 7, IL-22 serum levels significantly correlated with the MELD score.

The MELD score includes the laboratory parameters for creatinine, bilirubin and international normalized ratio for prothrombin time (INR). Therefore, we also investigated if the individual parameters of the MELD score correlate with serum IL-22 levels in our patients. As illustrated in Table 2, creatinine and INR but not bilirubin correlated with IL-22 serum levels.

**Table 2:** Correlation of the IL-22 serum concentration with hematological and biochemical parameters

Parameter	Spearman correlation, *r*	*P*-value
**Prognosis score**		
MELD	0.351	<0.001
Creatinine	0.271	0.004
Bilirubin	0.103	>0.2
INR	0.317	0.001

**Synthesis**		
Albumin	-0.222	0.028

**Acute phase**		
CRP	0.432	<0.001

**Liver enzymes**		
AST	-0.093	>0.2
ALT	-0.187	0.047

**Tumor marker**		
AFP	0.018	>0.2

### IL-22 serum levels correlate with surrogate parameters for inflammation

To investigate whether IL-22 serum levels are associated with determinants of liver synthetic capacity, inflammation or damage, potential correlations of the cytokine with serum albumin (surrogate marker of liver synthetic capacity), C-reactive protein (CRP, surrogate marker of ongoing inflammation), and alanine aminotransferase (ALT) as well as aspartate aminotransferase (AST), both surrogate markers of liver damage, were analyzed (Table 2). A strong positive correlation was found between serum IL-22 and CRP levels (Table 2). Furthermore, weak but significant inverse correlations between serum levels of IL-22 and albumin, as well as ALT, were observed (Table 2).

### Elevated IL-22 serum levels are predictive for reduced survival independently from age, CRP, creatinine, MELD score and presence of liver-related complications

In addition to MELD score and elevated IL-22, the factors of high CRP (≥2.9 mg/dl) [28], elevated serum creatinine, presence of liver-related complications, and age (*P *= 0.005, HR 0.314, CI (0.141 to 0.702); *P *= 0.05, HR 0.453, CI (0.203 to 1.012); *P *= 0.028, HR 0.258, CI (0.077 to 0.862); *P *= 0.011, HR 0.955, CI (0.922 to 0.989)) were associated with reduced overall survival in univariate analysis. Age-adjusted multivariate Cox proportional-hazards analysis (including all significant variables in univariate analysis) identified elevated systemic IL-22 levels as independent predictors of reduced survival (*P *= 0.007, HR 0.218, CI (0.072 to 0.662) for IL-22; *P *= 0.005, HR 0.93, CI (0.89 to 0.98) for age). High CRP, elevated creatinine, presence of liver-related complications, and MELD score ≥20 were not predictive for survival in multivariate analysis (*P *= 0.077, *P *> 0.2, *P *= 0.136 and *P *= 0.069, respectively).

We, furthermore, performed an AUC analysis to compare MELD score and IL-22 levels for the ability to discriminate patients who died during follow-up and those who did not. In this analysis, serum IL-22 significantly discriminated patients who died during the follow-up period from those who survived (AUC 0.682, CI (0.560 to 0.805), *P *= 0.010). In contrast, the MELD score did not identify patients who died during the follow-up period (AUC 0.611, CI (0.470 to 0.752), *P *= 0.118).

## Discussion

The role of IL-22 in disease progression of patients with liver cirrhosis is currently uncertain. Lymphoid cells are supposed to be the major source of IL-22 [3], though cells of the macrophage/dendritic cell type have been likewise reported to be capable of producing the cytokine [14,29]. In contrast, major liver targets of IL-22 are certainly hepatocytes which express functional IL-22 receptors to a large extent [22]. In the present study, we performed immunohistochemistry to confirm IL-22 expression in cirrhotic livers. Indeed, IL-22 was detectable in non-parenchymal cells in the hepatic lobe and in areas of necroinflammation. These observations are in agreement with previous data from patients and murine models, suggesting that the liver is an important source of serum IL-22 under conditions of hepatic inflammation/injury [20,26].

The potential role of IL-22 in liver diseases has been intensively studied in murine models for T cell-mediated hepatitis [20], fulminant hepatic failure [30], alcoholic liver injury [22] and regeneration after hepatectomy [31]. In those models, IL-22 attenuated liver injury [20,22], prevented hepatic failure [30] and improved hepatic steatosis [22]. On the other hand, blockage of IL-22 bioactivity increased liver injury [20] and was associated with decreased hepatocyte proliferation following hepatectomy [31]. On the whole, with the exception of experimental hepatitis B virus infection [24], murine models largely suggest a tissue protective function of IL-22 in hepatic disorders.

The pathophysiological relevance and prognostic potential of serum IL-22 in patients with liver diseases of various etiologies is less clear. The limited data available suggest increased serum IL-22 in patients with acute HBV infection [24] and in patients with chronic hepatitis [23], respectively. This latter study also links hepatocarcinogenesis to IL-22 function. IL-22 may be particularly important for outcome of liver cirrhosis. To relate systemic IL-22 to the prognosis of clinical liver cirrhosis, we performed a prospective cohort study in which patients with advanced liver cirrhosis were consecutively enrolled and longitudinally followed. Our data show that, compared to healthy donors, IL-22 serum levels were significantly elevated in patients with liver cirrhosis. Furthermore, elevated IL-22 levels were associated with liver-related complications, such as ascites, hepatorenal syndrome and spontaneous bacterial peritonitis. These observations altogether indicate that high IL-22 serum levels may reflect the severity of liver disease.

IL-22 sera contents in healthy donors were, for the most part, barely detectable and set the basis for calculation of a reference range. This reference range defined levels below 18 pg/ml as being normal (ULN), which agrees with previous reports on IL-22 in sera of healthy donors obtained in the US and Europe [6,9,27,32]. According to this threshold, 47.5% of patients with liver cirrhosis showed elevated IL-22 serum concentrations. Follow-up analyses of serum IL-22 levels in patients with liver cirrhosis suggest that mean IL-22 levels increase during the course of liver disease. The majority of patients with elevated IL-22 baseline levels maintain elevated levels during follow-up, while more than half of patients with normal IL-22 serum levels at baseline develop increased levels during follow-up. These results indicate that IL-22 elevation is not a transient phenomenon in patients with liver cirrhosis. The mechanisms mediating this IL-22 increase in the patients are not yet clear. However, it can be assumed that increasing IL-22 levels are connected with increased cytokine production as well as reduced hepatic or renal elimination.

Up-regulation of IL-22 might be associated with the underlying etiology of liver cirrhosis. In the present study, however, we observed no difference in the IL-22 levels in sera from patients with prevalent etiologies of liver disease, that is, HBV, HCV and alcoholic liver cirrhosis. In the course of this study, hepatocellular carcinoma was diagnosed in two patients with detectable IL-22. This observation is in line with elevated systemic IL-22 in patients with hepatocellular carcinoma [23].

Advanced liver cirrhosis is associated with poor prognosis. Accordingly, prediction of hepatic deterioration is key to efficient management of the clinical disease. In the present study, approximately one quarter of patients died after one year due to hepatic deterioration. The primary goal of this study was to investigate whether serum IL-22 relates to survival of patients with liver cirrhosis. In fact, serum IL-22 was significantly associated with survival. However, patients with elevated serum IL-22 (>18 pg/ml) levels showed reduced survival compared to those patients with normal IL-22 levels indicating that processes leading to deterioration of liver cirrhosis and its sequelae come along with an increase of serum IL-22. With respect to the hepatoprotective effects of IL-22 in animal models, we assume that elevated IL-22 serum levels in liver cirrhosis still serve protective functions. However, it cannot be excluded that IL-22 beyond a certain threshold may be pathogenic.

The best currently evaluated prognosis score for patients with liver cirrhosis is the MELD score. Systemic IL-22 levels in patients with liver cirrhosis significantly correlated with the MELD score, substantiating that IL-22 is associated with deterioration of liver function and subsequent mortality of cirrhotic patients. The MELD score is a short-term (three- to six-month) predictor of survival in patients with end-stage liver disease, but is a weak predictor of survival in patients with compensated liver cirrhosis in the long term. In multivariate analysis, systemic IL-22 was (independently from age, presence of liver-related complications, elevated creatinine, high CRP and high MELD score) associated with long-term mortality. Taking into account that IL-22 serum levels were stably increased in the majority of patients in the course of liver cirrhosis, the IL-22 serum level may be a valuable prognostic marker for long-term survival of patients with advanced liver cirrhosis.

The MELD score includes three blood surrogate parameters addressing different aspects of liver deterioration. INR and bilirubin reflect liver synthetic capacity and excretory function, while creatinine indicates renal decompensation due to hepatic failure. IL-22 serum levels correlated with two parameters of the MELD score, that is, creatinine and INR. Furthermore, weak inverse correlations were observed between systemic IL-22 and serum albumin and ALT. The weak but significant inverse correlation between the ALT level and IL-22 serum level must be carefully interpreted, but may indicate that IL-22 has a hepatoprotective function in patients with advanced liver cirrhosis.

IL-22 also correlated with CRP, a well-established surrogate marker of hepatic inflammation and prognosis of liver cirrhosis [28]. CRP and creatinine were both associated with serum IL-22, further suggesting that enhanced systemic IL-22 is driven by hepatic inflammation along with renal deterioration. Whether IL-22 bioactivity likewise contributes to renal deterioration is unknown

In contrast to the surrogate parameters currently used for the assessment of the prognosis of liver cirrhosis, IL-22 likely plays an active role in liver inflammation, regeneration and carcinogenesis [20,22,30,31]. Of particular interest is the association among serum IL-22, CRP and spontaneous bacterial peritonitis, indicating that IL-22 production is augmented by the infection. In fact, previous studies showed up-regulation of the systemic IL-22 level in patients with abdominal sepsis [9].

## Conclusions

We demonstrate herein that elevated systemic IL-22 levels are predictive for reduced survival in patients with liver cirrhosis independent of age, presence of liver-related complications, CRP, creatinine and the MELD score. Our data indicate that processes in the liver that lead to deterioration of liver cirrhosis and its sequelae are associated with an increase of IL-22. Overproduction of IL-22 in liver cirrhosis may serve protective functions as indicated by murine models of disease; however, IL-22 beyond a certain threshold may also be pathogenic.

## Abbreviations

AC: alcoholic cirrhosis; ALT: alanine aminotransferase; AST: aspartate aminotransferase; AUC: area under the observed receiver operating curve; CRP: C-reactive protein; HBV: chronic hepatitis B virus; HCV: chronic hepatitis C virus; HRS: hepatorenal syndrome; IL-22: interleukin-22; INR: international normalized ratio; MELD: model of end stage liver disease; NK: natural killer; NO: nitric oxide; ULN: upper limit of normal

## Competing interests

The authors declare that they have no competing interests.

## Authors' contributions

BK, AP and HM initiated and conceived the study, analyzed the data and drafted the manuscript. IR and MB performed the immunoassays and were involved in drafting the manuscript. FB and LK collected the data and blood samples and were involved in planning the study. NF, OW and EH participated in the design of the study and performed statistical analyses. JP and SZ were involved in study design, analysis of the data and critical revision of the manuscript. All authors read and approved the final manuscript.

## Authors' information

BK, OW and SZ are specialists in hepatology with a main focus on viral hepatitis, liver cirrhosis and liver transplantation. JP, AP and HM are scientists with a focus on signal transduction and cytokine biology. MB is a staff scientist. NF and EH are mathematicians and statisticians with a special interest in mathematical modeling. FB, LK and IR are doctoral students.

## Pre-publication history

The pre-publication history for this paper can be accessed here:

http://www.biomedcentral.com/1741-7015/10/102/prepub
